# The interplay between aerobic metabolism and antipredator performance: vigilance is related to recovery rate after exercise

**DOI:** 10.3389/fphys.2015.00111

**Published:** 2015-04-09

**Authors:** Shaun S. Killen, Donald Reid, Stefano Marras, Paolo Domenici

**Affiliations:** ^1^Institute of Biodiversity, Animal Health and Comparative Medicine, College of Medical, Veterinary and Life Sciences, University of GlasgowGlasgow, UK; ^2^Institute for the Coastal Marine Environment (IAMC), National Research Council (CNR)Oristano, Italy

**Keywords:** metabolic rate, aerobic scope, predator–prey interactions, swimming, teleost fish

## Abstract

When attacked by a predator, fish respond with a sudden fast-start motion away from the threat. Although this anaerobically-powered swimming necessitates a recovery phase which is fueled aerobically, little is known about links between escape performance and aerobic traits such as aerobic scope (AS) or recovery time after exhaustive exercise. Slower recovery ability or a reduced AS could make some individuals less likely to engage in a fast-start response or display reduced performance. Conversely, increased vigilance in some individuals could permit faster responses to an attack but also increase energy demand and prolong recovery after anaerobic exercise. We examined how AS and the ability to recover from anaerobic exercise relates to differences in fast-start escape performance in juvenile golden gray mullet at different acclimation temperatures. Individuals were acclimated to either 18, 22, or 26°C, then measured for standard and maximal metabolic rates and AS using intermittent flow respirometry. Anaerobic capacity and the time taken to recover after exercise were also assessed. Each fish was also filmed during a simulated attack to determine response latency, maximum speed and acceleration, and turning rate displayed during the escape response. Across temperatures, individuals with shorter response latencies during a simulated attack are those with the longest recovery time after exhaustive anaerobic exercise. Because a short response latency implies high preparedness to escape, these results highlight the trade-off between the increased vigilance and metabolic demand, which leads to longer recovery times in fast reactors. These results improve our understanding of the intrinsic physiological traits that generate inter-individual variability in escape ability, and emphasize that a full appreciation of trade-offs associated with predator avoidance and energy balance must include energetic costs associated with vigilance and recovery from anaerobic exercise.

## Introduction

Numerous mechanisms have evolved that allow animals to escape predators at virtually all points along the sequence of a typical predator–prey encounter (Hart, 1997; Killen, 2011). For fish, a fast-start escape response occurs during the critical first few milliseconds of a predator attack. It is a brief, sudden, and anaerobically-powered acceleration by the prey, consisting of a unilateral muscle contraction in a direction opposite to the threat, followed by an additional contraction on the other side of the body to propel the fish further away from the predator (Domenici and Blake, 1997). Escape responses in fish are usually controlled by a pair of giant reticulospinal neurons in the hindbrain, the Mauthner cells (Eaton et al., 1977), although non-Mauthner cell responses are also possible (Kohashi and Oda, 2008). Such non-Mauthner escape responses, however, tend to show longer latencies between stimulus detection and the resulting escape attempt (Liu and Fetcho, 1999; Eaton et al., 2001; Kohashi and Oda, 2008). Responsiveness and locomotory performance during this response likely influences which individuals are capable of surviving a predator attack (Domenici, 2010), and shows repeatable variation among individuals of the same species (Marras et al., 2011). However, the factors that generate and maintain this variation remain largely unknown.

The ideal strategy for avoiding predators is a balance between fleeing too early, which can result in lost foraging opportunities, and fleeing too late, which can increase the risk of being captured (Ydenberg and Dill, 1986; Krause and Godin, 1996; Bohórquez-Herrera et al., 2013). The optimal position of this trade-off may vary among individuals in relation to intrinsic traits (Jones and Godin, 2010). For example, individual fish vary in the rate at which they physiologically recover from anaerobic exercise such as that used during fast-starts (Marras et al., 2010; Killen et al., 2014). The waste products from this process must subsequently be metabolized during a recovery phase which is fueled aerobically (Richards et al., 2002). This post-exercise rise in aerobic metabolism represents the energetic cost of the escape response, but will also occupy a proportion of the individual's aerobic scope (AS) until recovery is complete, possibly constraining performance of other oxygen-consuming physiological functions. Furthermore, although escape responses are fueled anaerobically, they are negatively affected by hypoxia (Domenici et al., 2007), suggesting that escape responses and oxygen needs are not completely decoupled. Overall, variation in recovery rate after anaerobic exercise among individual fish could affect the relative costs and benefits of engaging in a fast-start response. Fish that recover more slowly from anaerobic exercise may incur a greater realized cost when engaging in a fast-start escape response, particularly if they have a reduced capacity for locomotory performance or other aerobic physiological processes during recovery. They may therefore be more reluctant to react to a potential attack or react more slowly. Further, fish with a higher AS are able to recover from anaerobic exercise faster (Marras et al., 2010; Killen et al., 2014), and so it is possible that fish with a larger AS may be more able to incur the costs associated with recovery, and therefore show greater responsiveness or performance during an escape response. Conversely, it is also possible that fish that react sooner or move faster during an attack may show a slower rate of recovery after anaerobic exercise, if their recovery is delayed due to the energetic costs of vigilance. On a mass-specific basis, the brain is one of the most metabolically active organs in the body (Rolfe and Brown, 1997), and high alertness and preparedness to escape may therefore entail an increased energetic demand (Millidine et al., 2006). There may also be costs associated with the maintenance of sensory systems that allow for increased threat perception and responsiveness. Overall, any such increases in energetic demand could prolong the phase of physiological recovery after an escape attempt or any other form of anaerobic metabolism.

Environmental factors can also affect locomotory performance, and often, the degree of variation in performance traits observed among individuals (Killen et al., 2013). Temperature, for example, has a strong influence on the metabolic physiology and locomotory performance of ectotherms (Angilletta et al., 2002) and neural performance (Montgomery and Macdonald, 1990). The effect of temperature on the fast-start response of fish is variable among species (Wilson et al., 2010), but is known to affect escape responsiveness (Webb, 1978; Preuss and Faber, 2003). Response latency tends to decrease at higher temperatures as a result of the effect of temperature on the speed of nerve conduction. However, fish subject to acute high temperature treatments may increase their latency as found in heat shock experiments in which high temperatures yielded longer reaction distances (Webb and Zhang, 1994). An increase in temperature has been associated with an increase maximum velocity during the fast-start response (Beddow et al., 1995) as a result of an increase muscle power output (Wakeling, 2006), but there appears to be a threshold temperature beyond which performance declines (Johnston and Temple, 2002). Furthermore, thermal acclimation can result in some degree of compensation (Johnson and Bennett, 1995; Wakeling, 2006). Temperature also influences the degree of physiological stress incurred during anaerobic exercise and the rate of subsequent recovery (Suski et al., 2006). Finally, temperature has a profound effect on AS in fishes, with AS being highest at a species-specific optimum temperature and then reduced at temperature above or below this point (Claireaux and Lefrancois, 2007; Pörtner and Farrell, 2008). It is therefore possible that temperature could modulate any effects of physiological traits on the fast-start escape response.

In this study we examined how the ability to recover from anaerobic exercise relates to differences in fast-start escape performance among individual fish. Further, we examined how acclimation temperature might modulate such relationships. We studied these issues in juvenile golden gray mullet *Liza aurata*. The young of this species inhabits lagoon environments where they are frequently targeted by a range of piscine and avian predators. Specifically, we investigated two alternative hypotheses regarding the interplay between aerobic metabolism and the fast-start escape response: (1) The aerobic-scope driven hypothesis: that individuals which respond to a simulated attack sooner and with a higher level of performance are those that recover sooner after anaerobic exercise, or that have a higher AS (i.e., high AS allows for a low threshold for escape); and (2) The vigilance-driven hypothesis: that fish with fast reaction times and high escape performance are those that show longer recovery rates, because they are the most vigilant and therefore have higher energetic demands that prolong physiological recovery after anaerobic exercise. The results of this study will improve our understanding of how intrinsic traits and environmental factors interact to affect anti-predator behaviors, and specifically performance during escapes, among individual animals of the same species.

## Materials and methods

### Animals

Juvenile Golden gray mullet (*n* = 42) were captured from the wild (Stagno di Cabras, Sardinia, Italy) in January 2012. Once at the laboratory (IAMC-CNR, Oristano, Sardinia, Italy) they were held under a 12–12 h light/dark photoperiod in a large cylindrical tank (2 m diameter, 1.5 m water depth) supplied with re-circulating, filtered natural seawater at a constant temperature (20°C) for 2 weeks. After this period, three groups of 14 individuals each were transferred to circular 100-L tanks, whilst initially retaining environmental parameters. Final acclimation temperatures of either 18, 22, or 26°C were reached by changing the water temperature by 1°C per day. Fish were left 1 month at the final acclimation temperature before being used in the experiment. Fish in the holding tanks were fed daily with a maintenance ration consisting of dried feed pellets. Individuals were fasted for 36 h before use in experiments. At experimentation, fish were 12.8 ± 0.81 cm total length and 15.3 ± 0.48 g mass (mean ± S.E.).

The fish were held, and the non-lethal experiments were conducted, in accordance with the laws governing animal experimentation in Italy. The IAMC-CNR facility of Oristano, where the fish were held and the experiments performed, is recognized by the Italian Government as a certified facility for fish rearing and ecophysiological experimentation (D.lgs. 116/92, Decreto n° 136/2011-A).

### Experimental protocol

As described and defined below, all traits were measured on every individual. This includes the following metabolic traits: standard metabolic rate (SMR), routine metabolic rate (RMR), maximal metabolic rate (MMR), aerobic scope (AS), excess post-exercise oxygen consumption (EPOC), and recovery time (T_R_) after exhaustive exercise; and the fast-start components: response latency, maximum speed (U_max_), maximum acceleration (A_max_), and turning rate.

Fish were first transferred to a fast-start arena, and left undisturbed for 12 h before testing for the escape response (see below for details). At the end of the escape response test fish were removed from the escape test arena and inserted into a static respirometer to determine SMR and RMR. The following day, aerobic scope (AS; i.e., the capacity to supply oxygen for all aerobic tasks above maintenance, including swimming, digestion, and recovery from anaerobic exercise) was determined by obtaining the difference between MMR and SMR.

### Fast-start test

The experimental set-up comprised of a circular tank (100 cm diameter × 80 cm depth and 25 cm water depth), supplied with re-circulating seawater at the fish acclimation temperature. The escape response of the fish was induced by mechanical stimulation (Dadda et al., 2010). The stimulus was a PVC cylinder with a tapered downward end and an iron bolt at the opposite end (10 cm height, 2 cm diameter, and weighing 35 g). The stimulus was released by an electromagnet from a height of 150 cm above the water surface. To prevent visual stimulation before contact with the water surface, the stimulus was released into a vertical PVC tube (15 cm of diameter) ending 0.5 cm before the water surface. A mirror inclined at 45° was used to identify the exact time the stimulus entered into the water. Light was supplied by two 250 W spotlights. The escape response arena was covered by a black tarpaulin, to screen the fish from visual disturbance. A high speed camera (Casio EXILIM High Speed EX-FH100, Casio Computer Company Ltd., Japan) was positioned above the experimental tank and recorded the escape response at 240 Hz. The fish were startled only within a range of angles between 80° and 100° relative to the stimulus, and at a relatively fixed distance from the stimulus, of between 20 and 30 cm.

The following variables were analyzed according to Marras et al. (2011): (1) Responsiveness, i.e., the percentage of fish, of the total analyzed, that responded to the stimulation with an escape response after being stimulated; (2) Latency, defined as the time interval between when the stimulus broke the water surface and the first detectable escape movement of the fish; (3) Distance-time variables, evaluated within a fixed time (58 ms; Dadda et al., 2010) which approximately corresponded to the average duration of stage 1 and 2 of the fast start response of all fish considered for all tests (mean escape duration), including cumulative distance, U_max_, and A_max_; (4) Stage 1 turning rate, calculated as the angle between the segment joining the center of mass and the tip of the head, at the beginning and at the end of the stage 1, divided by the stage duration. A polynomial regression procedure with five smoothed moving points was then applied for each derivative procedure (i.e., speed and acceleration) as described by Lanczos (1956).

### Respirometry

Fish were removed from the escape response arena and placed in static respirometers (0.5 L, Loligo Systems, Denmark) immersed in an outer tank maintained at either 18, 22, or 26°C (as appropriate depending on the acclimation temperature for each fish. Instantaneous oxygen uptake (MO_2_, in mg O_2_ h^−1^) was measured by intermittent flow respirometry (Steffensen, 1989) once every 30 min. Temperature was kept constant for the duration of the experiment. Water flow from the external bath through the respirometers was driven by an external pump that was set to turn on and off for alternating 15-min periods. This allowed decreases in water oxygen content to be measured every 15 s for 15 min while the respirometer was in the closed state. The respirometer was then flushed with aerated water for 15 min. The oxygen consumption during each closed phase was calculated using linear least squares regression (excluding the first and last 2 min of each closed phase). Water oxygen levels were measured with optodes (Oxy-4 mini; PreSens Precision Sensing GmbH, Regensburg, Germany) and associated software (Pre-Sens Oxy 4v2). Fish remained in the chambers for approximately 24 h. Whole-animal SMR (in mg O_2_ h^−1^) was estimated as the lowest tenth percentile of measurements taken throughout the measurement period (Dupont-Prinet et al., 2010; Killen, 2014), excluding the first 2 h of confinement in the chambers during which oxygen consumption was often elevated. RMR was measured as the mean level of oxygen uptake during this time. Fish were then removed from the chambers, placed into a circular arena (100 cm diameter × 80 cm depth and 25 cm water depth) and manually chased until exhaustion until they were no longer responsive (Clark et al., 2013; Killen, 2014). They were then placed back into the respirometry chambers, and MO_2_ was measured as previously described for 5 h. Measurements of background microbial respiration in the system were taken before and after the oxygen measurement in the respirometers. The highest rate of MO_2_ measured during the 5 h period after exhaustive exercise was taken as the MMR of the fish, and almost always occurred during the first measurement period after exercise. This method for determining MMR assumes that maximal rates of oxygen uptake are achieved during the recovery from the bout of exhaustive anaerobic exercise (Reidy et al., 2000; Killen et al., 2007; Clark et al., 2013). Aerobic scope was calculated as the difference between MMR and SMR. Individual recovery time (T_R_) was assessed as the time required for oxygen uptake to return from the maximal post exercise MO_2_ to the level at which the animal had available 50% of its total AS (Marras et al., 2010; Killen et al., 2014). EPOC for each individual was estimated by calculating the area under the sixth-order polynomial recovery function, above RMR, until the time at which fitted values were equal to individual RMR (Killen et al., 2014). For some fish (12/42 fish, with only one fish predicted to take longer than 7 h to return to RMR), values never returned to RMR within the 5 h measurement period after exercise. In these cases EPOC was estimated using an extrapolation of the recovery function until the fitted values were equal to RMR. EPOC represents the increase in oxygen consumption above routine levels occurring during recovery from a bout of exhaustive anaerobic exercise and represents the anaerobic capacity of an animal (Gastin, 1994; Lee et al., 2003). Note that fish were always transferred between experimental setups without air exposure. At the end of the metabolic measurements, fish were removed from the respirometers and measured for total length and mass.

### Data and statistical analysis

All tests were performed using SPSS Statistics (v. 20). The effect of temperature on metabolic traits (i.e., SMR, RMR, MMR, AS, EPOC, and recovery time) was assessed using general linear models. Absolute vales for either SMR, RMR, MMR, AS, EPOC, or T_R_ were used as the dependent variable, with temperature as a categorical explanatory variable and body mass as a covariate. We then examined links among metabolic traits, temperature, and fast-start components using general linear models. In a given model, fast-start components (either of response latency, U_max_, A_max_, turning rate) were used as the dependent variable, and SMR, AS, EPOC, T_R_, body mass and temperature were included as explanatory variables. Interactions between each explanatory variable and temperature were removed when not significant and the models re-run.

## Results

Only four out of 42 fish were not responsive to the stimulus designed to elicit a fast-start escape response. Although all four non-responsive fish were among those acclimated to 26°C, the small number of fish that were non-responsive precluded statistical analyses of the factors affecting responsiveness.

**Figure 1:**
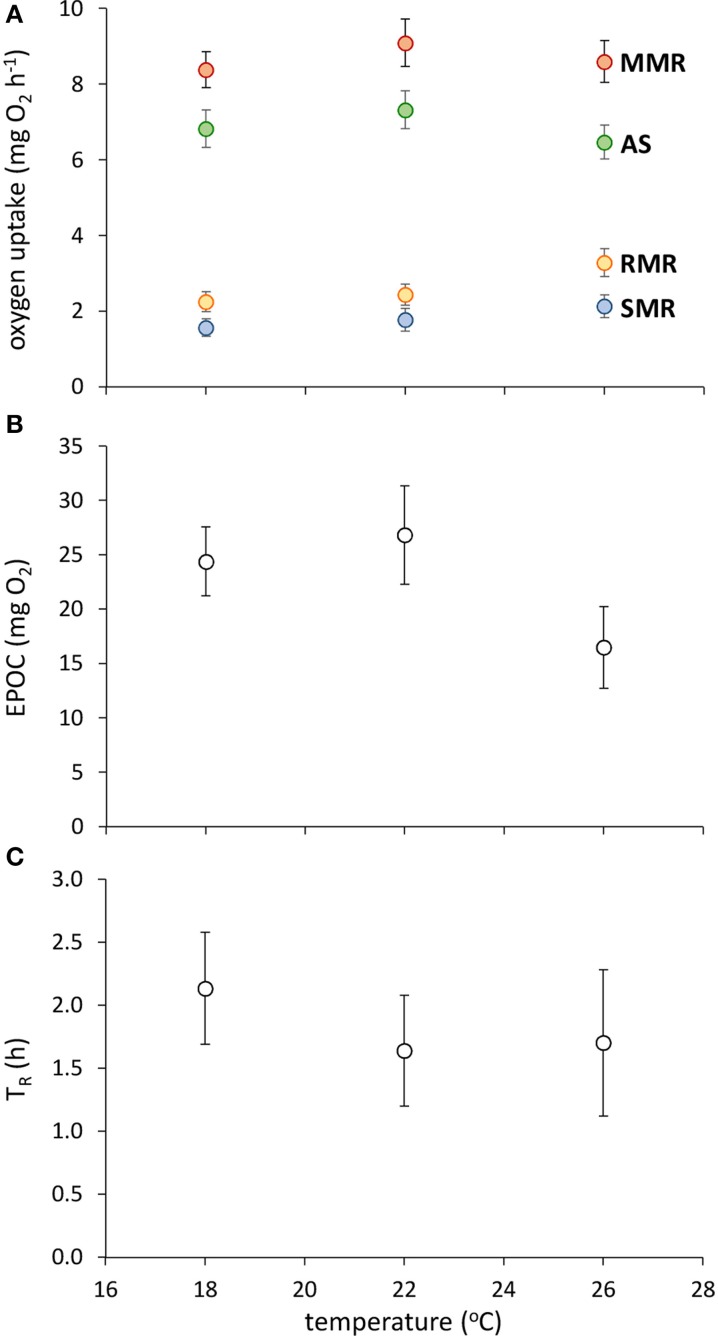
**Effect of temperature on metabolic traits in juvenile golden gray mullet. (A)** Traits associated with aerobic metabolism (SMR, standard metabolic rate; RMR, routine metabolic rate; AS, aerobic scope; MMR, maximal metabolic rate); **(B)** anaerobic capacity, as indicated by excess post-exercise oxygen consumption (EPOC); and **(C)** recovery rate after exhaustive exercise, as indicated by the time taken until recovery of 50% of total aerobic scope (T_R_). There was no significant effect of temperature on any response variable (see Results). Error bars = s.e.m; *n* = 14 fish per temperature.

Fish acclimated to warmer temperatures had a higher RMR [Figure 1; GLM, effect of temperature, *F*_(2,42)_ = 5.624, *p* = 0.007], but none of SMR, MMR, AS, EPOC, or T_R_ were influenced by acclimation temperature (Figure 1; GLM, effect of temperature, *p* > 0.05 in all cases).

**Figure 2:**
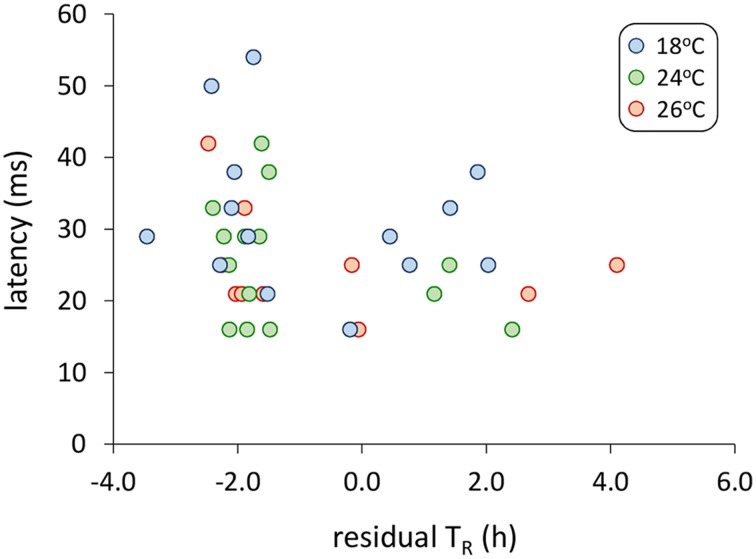
**Relationship between response latency during the fast-start escape response and time until 50% recovery after exhaustive exercise (T_R_)**. For this visual representation, T_R_ is shown as residual values after correction for variation in body mass, though uncorrected values were used in the general linear model analysis presented in Table 1, with body mass as a covariate. GLM analysis revealed a significant effect of T_R_ on response latency [Table 1; *F*_(1,37)_ = 7.06, *p* = 0.012]. *n* = 14 fish per temperature.

**Figure 3:**
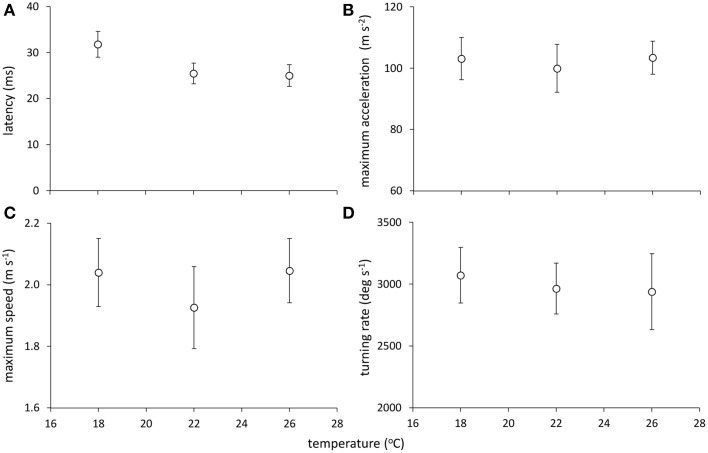
**Effect of temperature on components of the fast-start escape response in juvenile golden gray mullet: (A) response latency; (B) maximum acceleration; (C) maximum speed; and (D) turning rate**. There was no significant effect of temperature on any response variable (see Results). Error bars = s.e.m; *n* = 14 fish per temperature.

Models examining the simultaneous influence of temperature and metabolic traits on fast-start components showed that fish with a longer T_R_ showed shorter response latencies [Figure 2; Table 1; GLM, effect of T_R_, *F*_(1,37)_ = 7.064, *p* = 0.012]. Fish with a higher AS also had shorter response latencies [Table 1; GLM, effect of AS, *F*_(1,38)_ = 5.416, *p* = 0.027]. Neither of SMR, AS, EPOC, or T_R_ were related to any other component of the fast-start escape response (GLM, *p* > 0.05 in all cases). Acclimation temperature did not affect any of response latency, U_max_, A_max_, or turning rate (Figure 3; GLM, effect of temperature, *p* > 0.05 in all cases), and there were no significant interactions between temperature and any of SMR, AS, EPOC, or T_R_.

**Table 1:** **General linear model results for the effects of body mass, standard metabolic rate (SMR), aerobic scope (AS), excess post-exercise oxygen consumption (EPOC), recovery time after exhaustive exercise (T_R_), and temperature on latency during the fast start escape response in juvenile golden gray mullet**.

**Term**	***df***	***F***	***p***	**Estimate**	**Lower 95% CI**	**Upper 95% CI**	***t***
Mass	1	2.96	0.095	1.145	−0.213	2.504	1.722
SMR	1	0.01	0.931	0.145	−3.226	3.516	0.088
AS	1	5.42	0.027	−2.841	−5.333	−0.348	−2.327
EPOC	1	3.39	0.076	0.005	−0.001	0.010	1.841
T_R_	1	7.06	0.012	−3.120	−5.518	−0.723	−2.658
Temperature (26)	2	1.586	0.222	0			
(22)				−2.987	−11.119	5.144	−0.750
(18)				3.050	−5.484	11.583	0.730
Error	30						
Total	37						

*Interactions between temperature and SMR, AS, EPOC, and T_R_ were included in the original model but removed when not significant*.

## Discussion

Our results show that fish responding to the stimulation with a short latency (i.e., fast reactors) tend to show long recovery times after exhaustive exercise and high AS. These results contrast with the “aerobic-scope driven” hypothesis that fast recovering individuals would be more willing to engage in burst-type locomotory behaviors. However, the results support the alternate “vigilance-driven” hypothesis that fish with the lowest latencies incur higher costs (and therefore longer recovery times after exercise) because they are most vigilant. Our results show that the shortest latencies were around 15 ms, in line with those recorded in Mauthner-mediated responses of other species (Eaton et al., 2001), while the longest latencies observed were around 50 ms and may be non-Mauthner responses (Kohashi and Oda, 2008). Vigilance is known to decrease the response time in prey (Krause and Godin, 1996), and in our experiments, the occurrence of a short latency is suggestive of high vigilance which may produce a lower threshold for a fast (Mauthner-cell mediated) response. A 30 ms difference between the shortest and the longest latencies is likely to provide an anti-predator advantage to fast reactors, since life or death may be determined by a matter of a few milliseconds during the initial phases of an escape response (Catania, 2009). This is especially true in structurally complex environments where escaping to the predator's first attack can allow the prey to hide and avoid a second attack. Juvenile golden gray mullet spent a large proportion of the year in lagoons, which are highly turbid and therefore avoiding the first attack may be fundamental for survival, since high turbidity can allow gray mullet to move out of the predator's visual field.

Highly vigilant individuals are likely to spend more energy because of higher activity in the brain (Roulin, 2001), one of the most metabolically active organs in the body (Roland, 1993; Rolfe and Brown, 1997). In vertebrates, vigilance is known to require increased oxygen consumption (Moss et al., 1998) and fish with no access to shelter show high oxygen consumption, at least partly because of increased vigilance (Millidine et al., 2006). Therefore, it is possible that highly vigilant individuals would have a relatively high metabolic rate after the being startled, and this would reflect in the longer recovery rate. Interestingly, neither SMR nor RMR were related to latency among individual fish. This suggests that increased vigilance may not incur increased costs while an animal is at rest, but that the costs may be context-dependent and especially pronounced in threatening situations (e.g., in the minutes or hours after an attack) when vigilance may be highest. It is also noteworthy that anaerobic capacity (as estimated by EPOC) had no relationship with response latency, and fast-start performance indices such as U_max_ and A_max_ were not related to recovery rate or AS. This is additional evidence that it is not energy expenditure during the escape response itself, nor the capacity to perform anaerobic exercise that is causing the prolonged recovery times among fish that respond sooner after an attack. Together these results suggest that a full assessment of the trade-offs related to high vigilance may need to include, in addition to the cost of lost feeding opportunities (Lima and Dill, 1990), the metabolic cost of vigilance (Millidine et al., 2006), which can result in a longer recovery phase after escape attempts.

The negative correlation between response latency and T_R_ suggests a functional trade-off between adaptations that enhance vigilance and responsiveness and those that permit rapid recovery of function following anaerobic exercise. A possible regulating mechanism is baseline levels of circulating glucocorticoids, which may be elevated in fast reactors. Cortisol, for example, is known to increase alterness and vigilance, and consequently escape behavior and cognitive performance in general (Johnson et al., 1992). Furthermore, high cortisol levels are usually associated with increased oxygen consumption (Davis and Schreck, 1997) which is in line with the long recovery times after exhaustive exercise observed in fast reactors.

Fast reacting individuals were also those with a relatively high AS. This result can also be interpreted in light of the increased cost of vigilance of such individuals. Having a high AS provides fast reactors with a wider scope for activity (Claireaux and Lefrancois, 2007), and therefore allows them to engage in metabolically costly physiological states, such as that related to high alertness. Their high AS means that an excess aerobic capacity beyond that needed to support high alertness is largely sustainable. Another possibility is that fish with a higher AS may also be more willing to engage in burst-type locomotion, given that recovery from anaerobic exercise is powered anaerobically. However, unlike some previous studies (Marras et al., 2010; Killen et al., 2014), we did not observe a statistically significant link between AS and T_R_. Indeed, as previously discussed, it was the fish that responded slowest to the simulated attack that recovered fastest after exhaustive anaerobic exercise. Finally, it is conceivable that there was variability in the amount of exercise performed among individuals needed to achieve complete exhaustion, which was correlated with both the AS of individuals and their subsequent recovery time. Additional research could examine the effect of AS on recovery time after at a comparable level of anaerobic exercise intensity but below complete exhaustion.

The fact that the fastest responders are also the slowest to recover after anaerobic exercise suggests that, for these individuals, there is an additional cost associated with an early or premature flight response beyond lost foraging opportunities (Ydenberg and Dill, 1986). Anaerobic burst-type swimming causes a depletion of intramuscular glycogen, ATP, and phosphogen stores (Milligan, 1996; Kieffer, 2000). During recovery these fuels are replenished aerobically, a process which in fish is mainly fueled by lipid oxidation (Richards et al., 2002). Until recovery is complete, however, the ability to perform additional bursts of anaerobically-powered movement may be limited. Further, the rise in oxygen consumption that occurs during recovery from anaerobic exercise will occupy at least a portion of an animal's AS, which could constrain other behaviors and physiological functions until recovery is complete. Therefore, although the behavioral decision to engage in costly burst-type activities may be vital for escaping predation, this type of locomotion can also carry appreciable costs.

Our results also show that golden gray mullet are resilient to changes in thermal acclimation within the range of temperatures examined in the current study. In general, the AS of fish peaks at some intermediate temperature and declines with acclimation to warmer or cooler temperatures (Fry, 1971; Claireaux and Lefrancois, 2007). The relatively wide breadth of temperatures over which golden gray mullet are able to maintain a high AS is likely an adaptation to life in lagoon environments, which can show large seasonal and even daily temperature fluctuations. The robustness of the fast-start response to acclimation temperature may also indicate that the vulnerability of juvenile golden gray mullet to predators is relatively constant a range of temperatures spanning 8°C. Furthermore, although not significant, our result show that latency tends to decrease with temperature, in agreement with previous work (Webb, 1978), and the idea that reaction time decreases with temperature because of slower nerve conduction speeds. Future work could examine how the escape response is affected by acute shifts in temperature.

In conclusion, our results show that individuals that take longer to recover after anaerobic exercise have a shorter escape response latency during a predator attack. After accounting for variation in other metabolic traits, fish with a higher AS are those that respond fastest to an attack. These findings indicate that individual metabolic traits may underlie variation in escape ability within prey species. Further, links between fast start ability and traits such as recovery ability may play a role in determining when individuals should choose to flee in a potentially dangerous situation. Additional work is needed to examine the ways in which the costs of engaging in anaerobic burst-type activity affects the economics of fleeing, beyond the traditional focus on lost foraging opportunities.

## Author contributions

SK, DR, SM, and PD conceived the study idea and designed the experiments. SK, DR, and SM conducted the experiments. SK, DR, SM, and PD contributed to the analysis of the data and the writing of the manuscript.

### Conflict of interest statement

The authors declare that the research was conducted in the absence of any commercial or financial relationships that could be construed as a potential conflict of interest.
